# Effect of tiotropium and olodaterol on symptoms and patient-reported outcomes in patients with COPD: results from four randomised, double-blind studies

**DOI:** 10.1038/s41533-016-0002-x

**Published:** 2017-02-02

**Authors:** Gary T. Ferguson, Jill Karpel, Nathan Bennett, Emmanuelle Clerisme-Beaty, Lars Grönke, Florian Voß, Roland Buhl

**Affiliations:** 1 Pulmonary Research Institute of Southeast Michigan, 29255 West 10 Mile Road, Suite A, Farmington Mills, MI 48336 USA; 2 North Shore Medical Arts LLP, Great Neck, New York, NY USA; 3 Boehringer Ingelheim Pharmaceuticals Inc., Ridgefield, CT USA; 4 Boehringer Ingelheim Pharma GmbH & Co. KG, Ingelheim, Germany; 5 Pulmonary Department, Mainz University Hospital, Mainz, Germany

## Abstract

Chronic obstructive pulmonary disease is associated with significant morbidity and mortality. Trials of maintenance chronic obstructive pulmonary disease treatments focus on improvement in lung function and reductions in exacerbations, while patients are much more concerned about symptoms and health status. Our aim was to investigate the effects of tiotropium + olodaterol on patient-reported health outcomes, breathlessness and night-time rescue medication use in patients with chronic obstructive pulmonary disease, compared to placebo, tiotropium or olodaterol monotherapy. Two pairs of replicate, phase III studies of 12 (OTEMTO 1 + 2) and 52 weeks’ (TONADO 1 + 2) duration were evaluated, in which patients received either tiotropium + olodaterol 2.5/5 or 5/5 μg, tiotropium 2.5 or 5 μg, olodaterol 5 μg or placebo, all delivered once daily via Respimat inhaler. Patient-reported outcomes included breathlessness assessed by transition dyspnoea index focal score, health status assessed by St George’s Respiratory Questionnaire total score and night-time rescue medication use at 12 or 24 weeks. Outcomes from the pooled study data are reported. Overall, 1621 and 5162 patients were treated in the OTEMTO and TONADO trials, respectively. Significantly larger improvements in St George’s Respiratory Questionnaire and transition dyspnoea index focal scores were observed and a greater proportion of patients were responders to therapy (based on minimum clinically important differences in St George’s Respiratory Questionnaire and transition dyspnoea index) with tiotropium + olodaterol compared to either monotherapy or to placebo. Tiotropium + olodaterol 5/5 µg significantly reduced night-time rescue medication usage.

## Chronic obstructive pulmonary disease: Combined inhaler proves effective

Results from four in-depth studies show that a combined inhaler is very effective for treatment of moderate to severe chronic lung disease. Alleviating the symptoms of chronic obstructive pulmonary disease (COPD), particularly sleep disturbance, is crucial to enhancing patients’ quality of life. Gary Ferguson at the Pulmonary Research Institute of Southeast Michigan, together with other scientists across the USA and Germany, analysed data from four large-scale studies to evaluate the efficacy of STIOLTO Respimat, a combination of two bronchodilators—tiotropium, and olodaterol, which tackle airway obstruction and breathlessness, improving long-term lung function. They found that the new drug combination triggered significant improvements in patients’ quality of life and levels of breathlessness. Use of night-time rescue medication in patients on STIOLTO Respimat was considerably reduced. A greater number of patients responded positively to the combined inhaler than to monotherapy.

## Introduction

Chronic obstructive pulmonary disease (COPD) is characterised by progressive airflow limitation that is partially reversible at best and is associated with chronic inflammation. Almost all patients experience breathlessness, chronic cough and sputum production.^1^ In addition, impaired daily functioning, activity limitation and reduced health-related quality of life (QoL) can occur.^2^ Several factors have been shown to significantly affect QoL in patients with COPD, including age, sex and disease severity. Improving QoL is not only important for patients but is also associated with reduced medical costs.^3, 4^ Slowing deterioration in health status and symptoms may be as relevant to patient QoL as improvement and should be an important aim of COPD treatment.

Though airflow limitation is diagnostic of COPD and an important factor in patient disease risk, it does not necessarily correlate with patient symptoms or QoL. Indeed, QoL and symptomatic scores, measured by such instruments as the St George’s Respiratory Questionnaire (SGRQ), have been shown to be more closely associated with frequent exacerbations and hospital readmissions in patients with COPD.^5, 6^ Thus, assessment of patient-reported outcomes, which measure the impact of disease on the daily lives of patients, can be as important as objective measurements of airflow limitation in determining the benefit of treatment for patients with COPD. This is reflected by the Global initiative for chronic Obstructive Lung Disease (GOLD) classification of disease severity, which now includes patient-reported outcomes used in clinical practice such as breathlessness (modified Medical Research Council score) or the COPD Assessment Test score. Frequent use of rescue medication can be a sign of increased respiratory symptoms and changes in rescue medication use may be an indicator of the efficacy of a treatment in controlling symptoms in patients with COPD.

In the daily management of COPD, tiotropium is an established once-daily long-acting muscarinic antagonist maintenance treatment that has been shown to provide a broad range of long-term improvements in lung function, QoL, exacerbation risk and exercise capacity.^7–13^ Olodaterol is a once-daily long-acting β_2_-agonist (LABA) delivered via the Respimat inhaler, with high selectivity and fast onset of action for the treatment of COPD.^14–17^ The combination of tiotropium + olodaterol, which targets two different mechanisms of bronchodilation and is delivered via the Respimat, is approved in the USA, Canada, Australia and Europe, and has been extensively studied in a large phase III clinical trial programme.^18–21^

The impact of tiotropium + olodaterol on patient-reported outcomes has been evaluated and mean changes reported as primary end points in two sets of large, replicate, phase III trials—OTEMTO and TONADO. These studies differed in two significant aspects: the OTEMTO trials were active- and placebo-controlled studies of 12 weeks’ duration,^19^ whereas the TONADO studies were active-controlled 52-week studies;^18^ and enrolled patients had moderate to severe COPD (OTEMTO) or moderate to very severe COPD (TONADO). Combining data from these two sets of parallel trials and assessing meaningfully important changes in patient-reported outcomes using responder and deterioration analyses allows us to better identify the clinical effect of these therapies and the proportion of patients that may benefit.

By presenting data from these two sets of parallel trials side by side, we hope to better understand the impact of tiotropium + olodaterol treatment on outcomes that are of direct relevance to patients: in a large body of data; at two time points following 12 and 24 weeks of treatment; and across a range of disease severities. Furthermore, we present, for the first time, responder and deterioration analyses to better identify the clinical effect of these therapies and the proportion of patients that may benefit.

## Results

### Patient disposition and baseline characteristics

In the OTEMTO studies, 1623 and 1621 patients were randomised and treated, respectively. Discontinuation rates were highest in the placebo arm. In the TONADO studies, 5163 patients were randomised and 5162 were treated. The discontinuation rate was higher for patients treated with a monotherapy, compared to patients treated with combination therapy.

Baseline demographics were generally similar across treatment groups. The majority of patients were male. In the OTEMTO studies, 47% of patients were current smokers, compared to 37% in the TONADO studies; 64% of patients in OTEMTO and 50% in TONADO were moderate/GOLD 2, with 11% of patients classed as severe (GOLD 4) in the TONADO studies (Supplementary Table S1). Mean baseline dyspnoea index (BDI) focal scores and baseline SGRQ total scores were similar between treatment groups in the OTEMTO and TONADO studies (Supplementary Table S2).

### Breathlessness

**Fig. 1:**
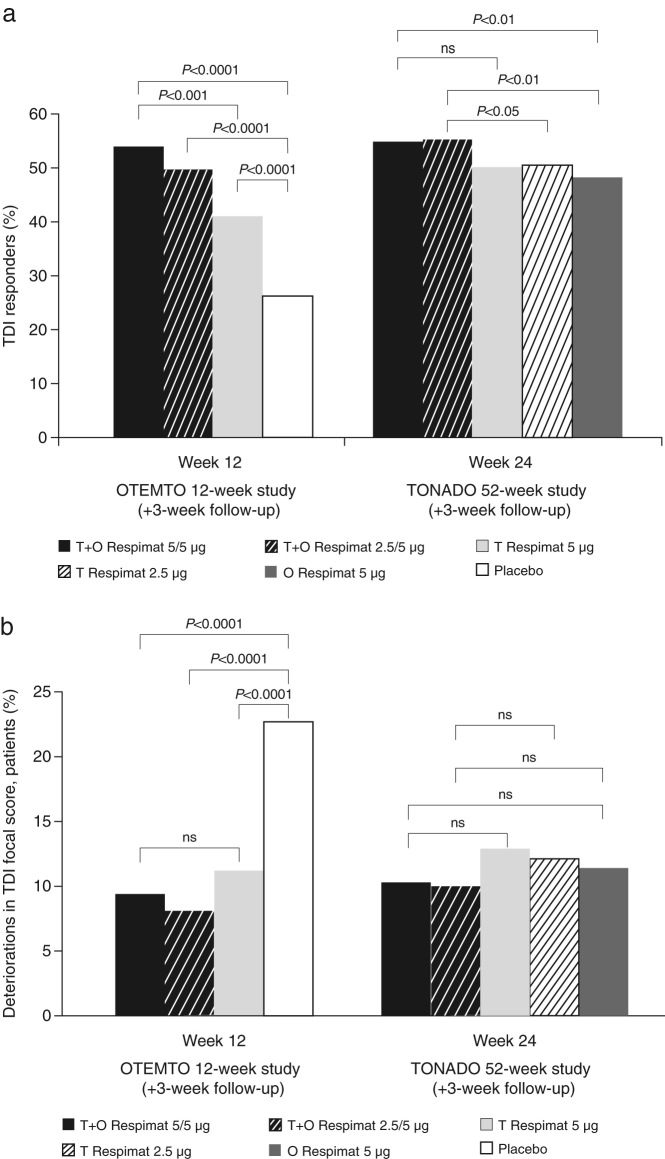
TDI responders (**a**) and deteriorators (**b**) after 12 weeks of treatment (OTEMTO) or 24 weeks of treatment (TONADO). ns, not significant; O, olodaterol; T, tiotropium; TDI, transition dyspnoea index

All treatments improved symptoms of breathlessness, assessed via transition dyspnoea index (TDI) scores. At both 12 and 24 weeks, there were greater percentages of TDI responders in the tiotropium + olodaterol arm than in the placebo or monotherapy arms. At week 12, 54% of patients receiving tiotropium + olodaterol 5/5 µg were classed as TDI responders compared to 41% receiving tiotropium (*P* < 0.001) and 26% of patients receiving placebo (*P* < 0.0001) in the OTEMTO studies. After 24 weeks of treatment, 55% of patients receiving tiotropium + olodaterol 5/5 µg were classed as TDI responders compared to 51% receiving tiotropium 5 µg (not significant; *P* = 0.0546) and 48% in the olodaterol 5 µg group (*P* < 0.01) in the TONADO studies (Fig. 1a; Supplementary Table S3).

Furthermore, analysis of TDI deteriorators showed that a significantly lower proportion of patients experienced TDI deterioration at week 12 in the OTEMTO studies with tiotropium + olodaterol (9%) or tiotropium monotherapy (11%) compared to placebo (23%; *P* < 0.0001 for both comparisons). At week 24, there was a low proportion of patients classed as TDI deteriorators and a lower number of patients receiving either dose of tiotropium + olodaterol were classed as TDI deteriorators (Fig. 1b, not significant; Supplementary Table S4). An analysis of the time to TDI deterioration over 52 weeks in TONADO showed a delayed onset of deterioration for tiotropium + olodaterol 5/5 µg with hazard ratios (95% confidence interval [CI]) of 0.82 (0.71, 0.96; *P* < 0.05) vs*.* olodaterol 5 µg and 0.84 (0.72, 0.98; *P* < 0.05) vs*.* tiotropium 5 µg.

**Fig. 2:**
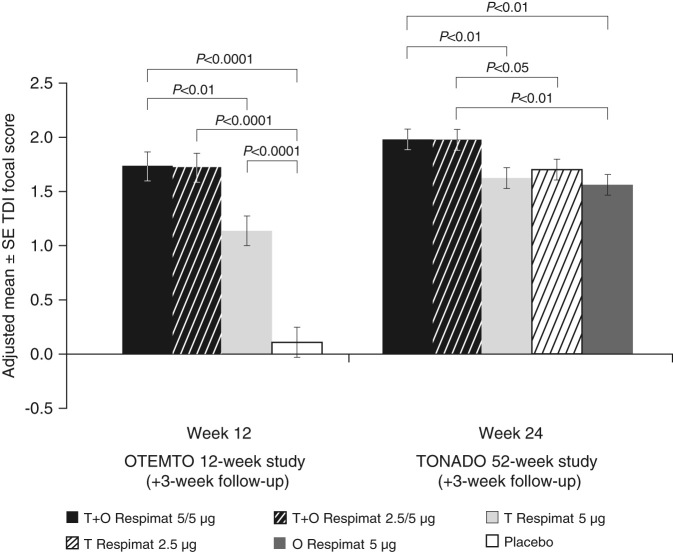
Adjusted mean TDI focal score after 12 weeks of treatment (OTEMTO) or 24 weeks of treatment (TONADO). O, olodaterol; SE, standard error; T, tiotropium; TDI, transition dyspnoea index

Analysis of TDI focal scores also showed that improvements in dyspnoea were greater in patients receiving tiotropium + olodaterol compared to either monotherapy or placebo in all studies. After 12 weeks of treatment in the OTEMTO studies, TDI focal scores improved by 1.62 units with tiotropium + olodaterol 5/5 µg compared to placebo (*P* < 0.0001) and 0.59 units compared to tiotropium 5 µg (*P* < 0.01). After 24 weeks of treatment in the TONADO studies, scores improved by 0.36 and 0.42 units with tiotropium + olodaterol 5/5 µg compared to tiotropium 5 µg (*P* < 0.01) and olodaterol 5 µg, respectively (*P* < 0.01) (Fig. 2; Supplementary Table S5).

### COPD-related health status

**Fig. 3:**
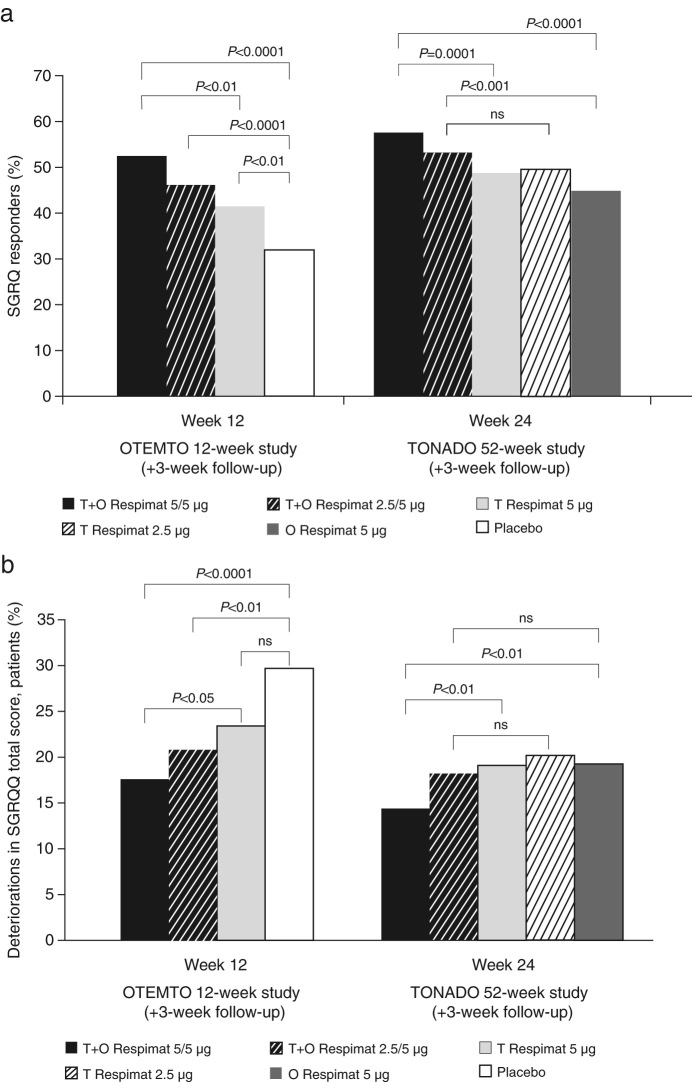
SGRQ responders (**a**) and deteriorators (**b**) after 12 weeks of treatment (OTEMTO) or 24 weeks of treatment (TONADO). ns, not significant; O, olodaterol; SGRQ, St George’s Respiratory Questionnaire; T, tiotropium

SGRQ total scores improved in all studies with tiotropium + olodaterol compared to the respective monotherapies or placebo. After both 12 and 24 weeks of treatment, there was a higher percentage of SGRQ responders in the groups receiving tiotropium + olodaterol 5/5 µg. After 12 weeks of treatment in the OTEMTO studies, 52% of patients receiving tiotropium + olodaterol were classed as SGRQ responders compared to 41% receiving tiotropium 5 µg (*P* < 0.01) and 32% in the placebo group (*P* < 0.0001). After 24 weeks of treatment in the TONADO studies, 58% of patients receiving tiotropium + olodaterol were classed as SGRQ responders compared to 49% receiving tiotropium 5 µg (*P* = 0.0001) and 45% receiving olodaterol 5 µg (*P* < 0.0001) (Fig. 3a; Supplementary Table S3).

Significantly fewer patients receiving tiotropium + olodaterol 5/5 µg were classed as SGRQ deteriorators (18%) compared to tiotropium 5 µg (23%; *P* < 0.05) or placebo (30%; *P* < 0.0001) at week 12 in OTEMTO. Similarly, at week 24, significantly fewer patients were classed as SGRQ deteriorators with tiotropium + olodaterol 5/5 µg (14%) compared to either monotherapy (19% for both; *P* < 0.01) in the TONADO studies (Fig. 3b; Supplementary Table S4). An analysis of the time to SGRQ deterioration over 52 weeks in TONADO showed a delayed onset of deteriorations for tiotropium + olodaterol 5/5 µg with hazard ratios (95% CI) of 0.70 (0.60, 0.82; *P* < 0.0001) vs*.* olodaterol 5 µg and 0.82 (0.70, 0.96; *P* < 0.05) vs*.* tiotropium 5 µg.

**Fig. 4:**
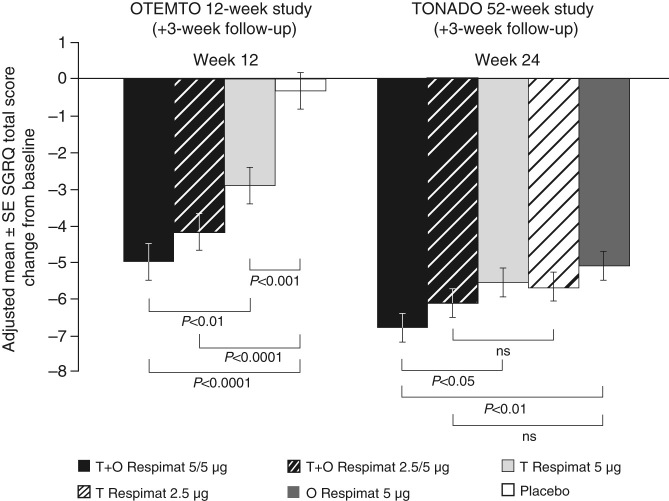
Change from baseline in adjusted mean SGRQ total score after 12 weeks of treatment (OTEMTO) or 24 weeks of treatment (TONADO). O, olodaterol; SE, standard error; SGRQ, St George’s Respiratory Questionnaire; T, tiotropium

Analysis of SGRQ total scores showed the largest reductions from baseline for patients receiving tiotropium + olodaterol 5/5 µg (Fig. 4), with a difference in SGRQ total score of 4.7 units with tiotropium + olodaterol 5/5 µg compared to placebo (*P* < 0.0001) and 2.1 units compared to tiotropium 5 µg monotherapy (*P* < 0.01) after 12 weeks of treatment (OTEMTO) (Supplementary Table S5). After 24 weeks of treatment, the largest reductions from baseline were also seen for patients receiving tiotropium + olodaterol 5/5 µg (Fig. 4); symptom scores were reduced by 1.2 units with tiotropium + olodaterol 5/5 µg compared to tiotropium 5 µg (*P* < 0.05) and 1.7 units compared to olodaterol 5 µg (*P* < 0.01) (TONADO) (Supplementary Table S5).

### Night-time rescue medication use

**Fig. 5:**
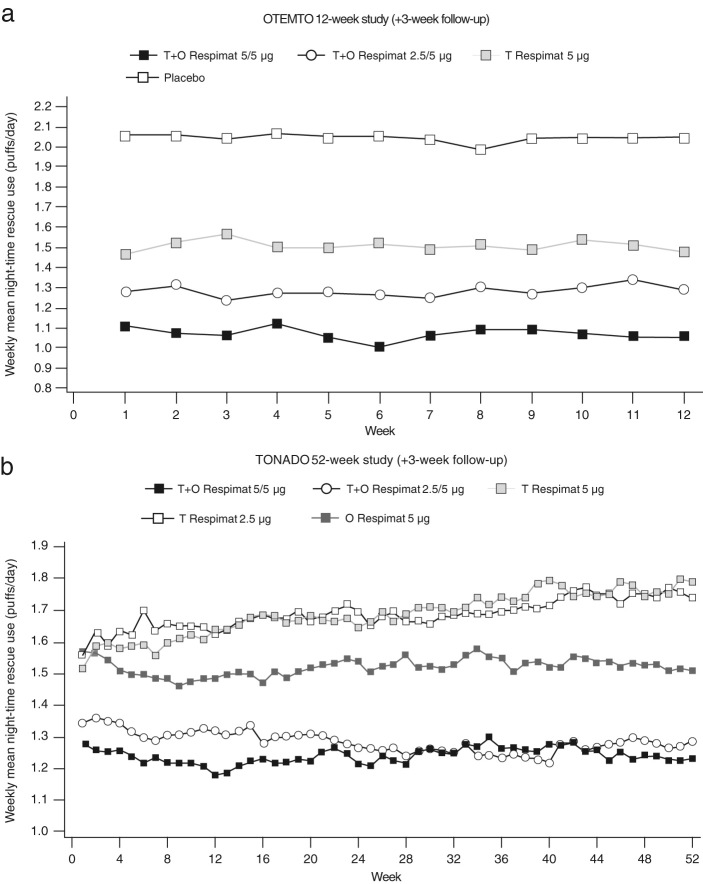
Adjusted weekly mean night-time rescue medication use over 12 weeks in the OTEMTO studies (**a**) and 52 weeks in the TONADO studies (**b**). O, olodaterol; T, tiotropium

Patients receiving tiotropium + olodaterol 5/5 µg required less rescue medication during the night over the 12-week period of the OTEMTO studies. Rescue medication use was reduced at week 12 by 0.42 puffs/night compared to tiotropium 5 µg (*P* < 0.001; 95% CI −0.64, −0.20) and by 1.00 puff/night compared to placebo (*P* < 0.0001; 95% CI −1.22, −0.78) (common baseline mean [standard error (SE)]: 2.01 [0.06]) (Fig. 5a).

These differences between treatment groups in night-time rescue medication use reductions were sustained, even when followed for 1 year in the TONADO studies, with adjusted mean night-time rescue medication use significantly reduced for patients receiving tiotropium + olodaterol by 0.32–0.43 puffs/night compared to monotherapy at 24 weeks (95% CI: tiotropium + olodaterol 5/5 µg vs*.* tiotropium 5 µg −0.58, −0.28, and −0.47, −0.17 vs*.* olodaterol 5 µg), and by 0.55 puffs/night compared to tiotropium 5 µg (*P* < 0.0001; 95% CI −0.72, −0.39) and 0.28 puffs/night compared to olodaterol 5 µg (*P* < 0.01; −0.44, −0.11) at 52 weeks (common baseline mean [SE] 2.20 [0.04]) (Fig. 5b).

### Safety

Incidence of adverse events (AEs) was similar across treatment groups in the OTEMTO studies, with more AEs leading to treatment discontinuation in the placebo groups compared to the treatment groups.^19^ In the TONADO studies, incidence of AEs was generally similar across treatment groups, ranging from 73–77% across treatment groups, with the majority being mild to moderate in severity. The majority of treatment-emergent AEs were respiratory events, particularly COPD exacerbations and infections.^18^

## Discussion

### Main findings

These studies demonstrated consistent improvements in patient-reported outcomes and night-time rescue medication use with once-daily tiotropium + olodaterol therapy, administered via the Respimat inhaler. Significantly greater numbers of patients were responders to therapy with tiotropium + olodaterol compared to the monotherapies and placebo, and a lower proportion of patients receiving tiotropium + olodaterol treatment experienced a worsening in their condition. In addition, significant improvements in health scores (SGRQ), breathlessness (TDI) and night-time rescue medication use for patients receiving tiotropium + olodaterol 5/5 µg were also seen compared to either tiotropium or olodaterol alone or placebo.

The minimum clinically important difference (MCID) for the SGRQ was reached between the tiotropium + olodaterol arm and placebo in the OTEMTO studies. Although MCID values have not been established for comparisons with an active comparator medication, there were significant improvements in SGRQ total scores and a significantly larger proportion of SGRQ responders, and lower proportion of SGRQ deteriorators (patients who achieved an improvement or worsening larger than the MCID, respectively) with tiotropium + olodaterol compared to the active comparators. This suggests that there is an improved, clinically relevant benefit for patients in QoL after treatment with tiotropium + olodaterol, compared to tiotropium or olodaterol alone.

Greater improvements over placebo in TDI focal scores with tiotropium + olodaterol were observed in the OTEMTO studies compared to the TONADO studies. As was observed for SGRQ scores, improvements with tiotropium + olodaterol compared to placebo reached the MCID for TDI focal score in OTEMTO. A greater proportion of patients receiving tiotropium + olodaterol were classed as TDI responders, according to MCID criteria, compared to tiotropium monotherapy in both studies. Furthermore, the proportion of patients who experienced a clinically relevant deterioration in TDI score was low in both studies, with significantly fewer patients receiving tiotropium + olodaterol classed as TDI deteriorators compared to placebo, and a longer time to deterioration, compared to either monotherapy. As TDI responder rates were similar for tiotropium + olodaterol between the OTEMTO and TONADO studies, but larger in TONADO for tiotropium monotherapy, these treatment differences only reached statistical significance in OTEMTO.

Night-time rescue medication use was reduced with tiotropium + olodaterol, compared to monotherapy in both studies, and compared to placebo in OTEMTO. Since patient habit is less likely to influence night-time rescue medication usage, measurement of this parameter may provide a better measure of symptom control. In addition, sleep disturbance is known to impact on QoL in patients with COPD and night-time rescue medication usage may be an important surrogate for night-time symptom control. Importantly, once medication education has been provided, rescue medication usage is a patient-reported outcome that indirectly evaluates a patient’s symptomatic status almost completely independently of doctor, nurse or other health-care professional contact.

### Interpretation of findings in relation to previously published work

The results of our trial are similar to those reported for other long-acting muscarinic antagonist/LABA combination treatments that have shown consistent improvements in lung-function and patient-reported outcomes, compared to placebo. Both once-daily dosing of indacaterol + glycopyrronium and umeclidinium + vilanterol have shown significant improvements in forced expiratory volume in 1 s (FEV_1_), SGRQ total score and TDI focal score compared to placebo after 26 and 24 weeks of treatment, respectively.^22, 23^ However, in contrast to tiotropium + olodaterol, neither of these other combinations showed consistent improvements when compared to the respective monotherapies. Indacaterol + glycopyrronium was superior to the respective monotherapies for lung-function outcomes but not SGRQ total score or TDI focal score. In comparison to tiotropium (18 µg) monotherapy, indacaterol + glycopyrronium and umeclidinium + vilanterol both showed superior improvements in FEV_1_, SGRQ total score and TDI focal score,^22, 24^ but little improvement over placebo was observed for tiotropium monotherapy in one study.^22^ However, these studies may have been limited by using open-label tiotropium for patients instead of blinded medications. Furthermore, no differences were seen between combined umeclidinium + vilanterol and tiotropium monotherapy in two other 6-month, phase III clinical studies.^25, 26^

Studies consistently show that improvements in lung function are associated with fewer symptoms and improved health status and QoL.^22, 23^

### Strengths and limitations of this study

The OTEMTO and TONADO studies presented here included a relatively broad range of patients with moderate to severe (OTEMTO) or moderate to very severe (TONADO) COPD and likely to require maintenance treatment for symptom management. These trials do have limitations. In the OTEMTO studies, patients with very severe (GOLD 4) COPD were excluded, as it was considered to be unethical for patients with very severe COPD to be randomised to placebo. In addition, the OTEMTO studies were conducted over a short duration (12 weeks). Nevertheless, results were broadly replicated in the longer TONADO trials, which did include patients with very severe COPD (11%). In the TONADO trials, the long duration of the study made inclusion of a placebo group unethical, limiting comparisons to active monotherapy treatments. Both sets of studies excluded patients with a significant disease other than COPD. This was defined as a disease that, in the opinion of the investigator, would put the patient at risk because of participation in the study, influence the results of the study or cause concern regarding the patient’s ability to participate in the study. However, the majority of patients in the TONADO and OTEMTO studies (>85% and >95%, respectively) had diagnosed co-morbidities at baseline, including cardiac and vascular disorders. Therefore, we consider the patients included to be broadly representative of those encountered in clinical practice.

In these studies, we used the BDI and corresponding TDI to assess improvements in breathlessness following active treatment. These are validated scales frequently used in clinical trials^27^ and the BDI has been found to significantly correlate with the modified Medical Research Council (mMRC) scale,^28^ which is more often used in clinical practice. Although the BDI/TDI and mMRC scales likely assess different elements of dyspnoea, we nevertheless show that, numerically, a greater proportion of patients experienced clinically relevant improvements in breathlessness with tiotropium + olodaterol compared to placebo or tiotropium monotherapy.

### Implications for future research, policy and practice

Symptoms of COPD are of particular relevance to patients and complement the lung-function outcomes commonly assessed in clinical trials. Outcomes that reflect symptoms, dyspnoea and health status may better assess the impact for patients of their disease on day-to-day functioning and their perception of treatment effects. Of particular relevance for patients are those symptoms, such as breathlessness or cough, that may lead to night-time awakenings. Sleep disturbance is both common and bothersome for patients and affects QoL.^29^ Finally, spirometry parameters may not adequately assess the impact of COPD and its co-morbidities on health-related QoL or relief from dyspnoea. Assessment of patient-reported outcomes, in conjunction with lung function, should help better evaluate the effectiveness of a treatment.^30^

## Conclusions

These data suggest that tiotropium + olodaterol once daily provides greater improvements, and reduces deterioration, in health-related QoL and dyspnoea, and provides greater improvements in night-time rescue medication use when compared to treatment with tiotropium alone, olodaterol alone or placebo for patients with moderate to very severe COPD. Tiotropium + olodaterol can provide benefits beyond improvements in lung function that are of relevance to the daily functioning of patients with COPD, including improvements in symptoms and overall health.

## Methods

### Study design

**Fig. 6:**
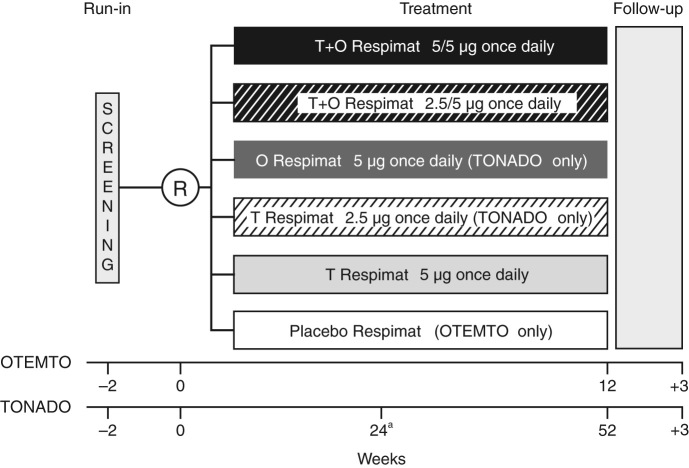
OTEMTO and TONADO study designs. O, olodaterol; R, randomisation; T, tiotropium. ^a^Primary end point assessment

Details of the methodology of the OTEMTO (1237.5 + 1237.6) and TONADO (1237.25 + 1237.26) studies have been published previously;^18, 19^ briefly, these were two sets of multinational, randomised, double-blind, parallel-group studies (registered with ClinicalTrials.gov: NCT01431274 [Study 1237.5], NCT01431287 [Study 1237.6], NCT01964352 [Study 1237.25] and NCT02006732 [Study 1237.26]). In the OTEMTO studies, patients received tiotropium + olodaterol 2.5/5 or 5/5 µg, tiotropium 5 µg or placebo, whereas in the TONADO studies, patients received tiotropium + olodaterol 2.5/5 or 5/5 µg, tiotropium 2.5 or 5 µg or olodaterol 5 µg (Fig. 6). Within the body of this manuscript, we have restricted our comparisons to the licensed dose of tiotropium + olodaterol 5/5 µg vs. tiotropium 5 µg, olodaterol 5 µg and placebo. However, for completeness, we have also included data for all active treatments within the figures and tables. All treatments were delivered via the Respimat inhaler.

### Patients

Patients were included if aged ≥40 years with a history of moderate to severe COPD (OTEMTO) (GOLD 2–3) or moderate to very severe COPD (TONADO) (GOLD 2–4), post-bronchodilator FEV_1_/forced vital capacity <70% and a smoking history of >10 pack-years. Exclusion criteria included: significant disease other than COPD; clinically relevant abnormal baseline laboratory parameters or a history of asthma; diagnosed thyrotoxicosis or paroxysmal tachycardia; history of myocardial infarction within 1 year of screening; unstable or life-threatening cardiac arrhythmia; hospitalised for heart failure within the past year; known active tuberculosis; life-threatening pulmonary obstruction; cystic fibrosis; clinically evident bronchiectasis; and previous thoracotomy with pulmonary resection or current enrolment in a pulmonary rehabilitation programme (or completed in the 6 weeks before screening).

In addition, in the OTEMTO studies, patients were excluded if they had suffered any COPD exacerbation or symptoms of lower respiratory tract infection within the previous 3 months. In the TONADO studies, patients were excluded if using daytime oxygen regularly and unable to abstain during clinic visits.

### Concomitant therapy

Inhaled corticosteroids could be continued during the studies providing the dose was stable for 6 weeks prior to screening. Long-acting muscarinic antagonists or LABAs other than study medication were prohibited during the screening or treatment periods, and short-acting muscarinic antagonists were permitted only during the screening period. Open-label salbutamol (OTEMTO) or salbutamol/albuterol metered-dose inhaler (100 μg per actuation) (TONADO) was provided as rescue medication for use throughout the studies. In the TONADO studies, temporary increases in the dose or addition of oral steroids or theophylline preparations were allowed during the treatment portion of the study; pulmonary function tests were not performed within 7 days of the last administered dose.

### Assessments

In the TONADO studies, SGRQ total score was a primary end point, completed by patients on day 1 (baseline value) and after 12, 24 and 52 weeks of treatment. Breathlessness was assessed via the BDI and TDI. These are interviewer-administered scales that relate dyspnoea to three domains of daily living: functional impairment, magnitude of task and magnitude of effort. The TDI at 24 weeks was a key secondary end point in the TONADO studies. Patients completed the BDI on day 1. TDI was then completed at 6, 12, 18, 24 and 52 weeks, immediately after the SGRQ assessment. Safety end points included AE reporting (recorded throughout the trial regardless of causality), vital signs, 12-lead electrocardiogram (pre-dose and repeated 40 min post-dose) and 24-h Holter monitoring in a sub-set of patients at selected sites.

In the OTEMTO studies, SGRQ total score was also a primary end point, completed at baseline then at weeks 6 and 12, prior to other assessments. TDI at 12 weeks was a secondary end point in the OTEMTO studies. The BDI was administered at baseline, with the TDI administered at weeks 6 and 12.

### Statistical analysis

SGRQ total score was analysed using combined data (OTEMTO 1 + 2 combined, TONADO 1 + 2 combined) using a restricted maximum likelihood-based mixed effects model repeated measures (MMRM) approach including the fixed, categorical effects of treatment, test day and treatment-by-test-day interaction, as well as the continuous fixed covariates of baseline and baseline-by-test-day interaction. A spatial power covariance structure was used to model the within-patient error. Patients were considered as a random effect. TDI focal score was analysed using the same MMRM approach. Rescue medication use was analysed as weekly mean number of puffs per day using analysis of covariance separately for each week, with a fixed categorical effect of treatment and baseline as a continuous covariate. For the responder and deteriorator analysis, a logistical regression, which included the fixed categorical effects of treatment, was used to calculate the odds ratio of responders between treatment groups. Time to first deterioration was analysed using a Cox proportional hazards model.

Further details can be found in the existing publications.^18, 19^ All studies were performed in accordance with the Declaration of Helsinki, the International Conference on Harmonisation Harmonised Tripartite Guideline for Good Clinical Practice and local regulations. Protocols were approved by the authorities and ethics committees of the respective institutions, and signed, informed consent was obtained from all patients.

### Study outcomes and assessments

In all studies, the TDI was performed by trained clinic staff,^31, 32^ assessing change in the amount and extent to which breathlessness limits activity levels. An increase of ≥1 unit from baseline on the TDI scale is considered clinically relevant^27^ and was used to define TDI responders. TDI deteriorators were defined as those patients with a decrease in TDI focal score ≥1 unit from baseline.

SGRQ total score was assessed at 12 weeks in OTEMTO and at 24 weeks in TONADO, assessing patients’ current health state, including symptoms, activity levels and impact on daily functioning. Patients were classed as responders or deteriorators if SGRQ total score was decreased or increased by ≥4 units from baseline, respectively, based on the proposed MCID for SGRQ.^33^

In all studies, rescue medication use, including night-time rescue medication use, was recorded by the patient in an e-diary. AEs were recorded throughout the study.

## Electronic supplementary material

Supplementary Material

## References

[1] Global Initiative for Chronic Obstructive Lung Disease. Global strategy for the diagnosis, management, and prevention of chronic obstructive pulmonary disease. Updated 2016. Last accessed on 27 April 2016. Available at http://www.goldcopd.org/.

[2] Cavaillès A, Brinchault-Rabin G, Dixmier A, Goupil F, Gut-Gobert C, Marchand-Adam S (2013). Comorbidities of COPD. Eur. Respir. Rev..

[3] Wu M, Zhao Q, Chen Y, Fu C, Xu B (2015). Quality of life and its association with direct medical costs for COPD in urban China. Health Qual. Life Outcomes.

[4] Wacker ME, Hunger M, Karrasch S, Heinrich J, Peters A, Schulz H (2014). Health-related quality of life and chronic obstructive pulmonary disease in early stages – longitudinal results from the population-based KORA cohort in a working age population. BMC Pulm. Med..

[5] Osman LM, Godden DJ, Friend JAR, Legge JS, Douglas JG (1997). Quality of life and hospital re-admission in patients with chronic obstructive pulmonary disease. Thorax.

[6] Seemungal TAR, Donaldson GC, Paul EA, Bestall JC, Jeffries DJ, Wedzicha JA (1998). Effect of exacerbation on quality of life in patients with chronic obstructive pulmonary disease. Am. J. Respir. Crit. Care Med..

[7] Casaburi R, Mahler DA, Jones PW, Wanner A, San Pedro G, ZuWallack RL (2002). A long-term evaluation of once-daily inhaled tiotropium in chronic obstructive pulmonary disease. Eur. Respir. J..

[8] O’Donnell DE, Flüge T, Gerken F, Hamilton A, Webb K, Aguilaniu B (2004). Effects of tiotropium on lung hyperinflation, dyspnoea and exercise tolerance in COPD. Eur. Respir. J..

[9] Maltais F, Hamilton A, Marciniuk D, Hernandez P, Sciurba FC, Richter K (2005). Improvements in symptom-limited exercise performance over 8 h with once-daily tiotropium in patients with COPD. Chest.

[10] Tashkin DP, Celli B, Senn S, Burkhart D, Kesten S, Menjoge S (2008). A 4-year trial of tiotropium in chronic obstructive pulmonary disease. N. Engl. J. Med..

[11] Bateman ED, Tashkin D, Siafakas N, Dahl R, Towse L, Massey D (2010). A one-year trial of tiotropium Respimat plus usual therapy in COPD patients. Respir. Med..

[12] Yohannes AM, Willgoss TG, Vestbo J (2011). Tiotropium for treatment of stable COPD: a meta-analysis of clinically relevant outcomes. Respir. Care.

[13] Vogelmeier C, Hederer B, Glaab T, Schmidt H, Rutten-van Mölken MPMH, Beeh KM (2011). Tiotropium versus salmeterol for the prevention of exacerbations of COPD. N. Engl. J. Med..

[14] Ferguson GT, Feldman GJ, Hofbauer P, Hamilton A, Allen L, Korducki L (2014). Efficacy and safety of olodaterol once daily delivered via Respimat in patients with GOLD 2–4 COPD: results from two replicate 48-week studies. Int. J. Chron. Obstruct. Pulmon. Dis..

[15] Koch A, Pizzichini E, Hamilton A, Hart L, Korducki L, De Salvo MC (2014). Lung function efficacy and symptomatic benefit of olodaterol once daily delivered via Respimat versus placebo and formoterol twice daily in patients with GOLD 2-4 COPD: results from two replicate 48-week studies. Int. J. Chron. Obstruct. Pulmon. Dis.

[16] Feldman GJ, Bernstein JA, Hamilton A, Nivens MC, Korducki L, LaForce C (2014). The 24-h FEV_1_ time profile of olodaterol once daily via Respimat and formoterol twice daily via Aerolizer in patients with GOLD 2–4 COPD: results from two 6-week crossover studies. Springerplus.

[17] Lange P, Aumann J-L, Hamilton A, Tetzlaff K, Ting N, Derom E (2014). The 24 hour lung function time profile of olodaterol once daily versus placebo and tiotropium in patients with moderate to very severe chronic obstructive pulmonary disease. J. Pulm. Respir. Med..

[18] Buhl R, Maltais F, Abrahams R, Bjermer L, Derom E, Ferguson G (2015). Tiotropium and olodaterol fixed-dose combination *versus* mono-components in COPD (GOLD 2–4). Eur. Respir. J..

[19] Singh D, Ferguson GT, Bolitschek J, Grönke L, Hallmann C, Bennett N (2015). Tiotropium + olodaterol shows clinically meaningful improvements in quality of life. Respir. Med..

[20] Beeh K-M, Westerman J, Kirsten A-M, Hébert J, Grönke L, Hamilton A (2015). The 24-h lung-function profile of once-daily tiotropium and olodaterol fixed-dose combination in chronic obstructive pulmonary disease. Pulm. Pharmacol. Ther..

[21] Beeh K-M, Derom E, Echave-Sustaeta J, Grönke L, Hamilton A, Zhai D (2016). The lung function profile of once-daily tiotropium and olodaterol via Respimat is superior to that of twice-daily salmeterol and fluticasone propionate via Accuhaler (ENERGITO study. Int. J. Chron. Obstruct. Pulmon. Dis..

[22] Bateman ED, Ferguson GT, Barnes N, Gallagher N, Green Y, Henley M (2013). Dual bronchodilation with QVA149 *versus* single bronchodilator therapy: the SHINE study. Eur. Respir. J..

[23] Donohue JF, Maleki-Yazdi MR, Kilbride S, Mehta R, Kalberg C, Church A (2013). Efficacy and safety of once-daily umeclidinium/vilanterol 62.5/25 mcg in COPD. Respir. Med..

[24] Maleki-Yazdi MR, Kaelin T, Richard N, Zvarich M, Church A (2014). Efficacy and safety of umeclidinium/vilanterol 62.5/25 mcg and tiotropium 18 mcg in chronic obstructive pulmonary disease: results of a 24-week, randomized, controlled trial. Respir. Med..

[25] European Medicines Agency. Anoro summary of product characteristics. Updated June 2014. Last accessed on 9 March 2016. Available at http://www.ema.europa.eu/ema/index.jsp?curl=pages/medicines/human/medicines/002751/human_med_001754.jsp&mid=WC0b01ac058001d124.

[26] Decramer M, Anzueto A, Kerwin E, Kaelin T, Richard N, Crater G (2014). Efficacy and safety of umeclidinium plus vilanterol versus tiotropium, vilanterol, or umeclidinium monotherapies over 24 weeks in patients with chronic obstructive pulmonary disease: results from two multicentre, blinded, randomised controlled trials. Lancet Respir. Med..

[27] Witek TJ, Mahler DA (2003). Minimal important difference of the transition dyspnoea index in a multinational clinical trial. Eur. Respir. J..

[28] Perez T, Burgel PR, Paillasseur J-L, Caillaud D, Deslée G, Chanez P (2015). Modified medical research council scale vs baseline dyspnea index to evaluate dyspnea in chronic obstructive pulmonary disease. Int. J. Chron. Obstruct. Pulmon. Dis..

[29] Agusti A, Hedner J, Marin JM, Barbé F, Cazzola M, Rennard S (2011). Night-time symptoms: a forgotten dimension of COPD. Eur. Respir. Rev..

[30] van der Molen T, Cazzola M (2012). Beyond lung function in COPD management: effectiveness of LABA/LAMA combination therapy on patient-centred outcomes. Prim. Care Respir. J..

[31] Mahler DA (2006). Mechanisms and measurement of dyspnea in chronic obstructive pulmonary disease. Proc. Am. Thorac. Soc..

[32] Mahler DA, Weinberg DH, Wells CK, Feinstein AR (1984). The measurement of dyspnea. Contents, interobserver agreement, and physiologic correlates of two new clinical indexes. Chest.

[33] Jones PW (2014). Estimation and application of the minimum clinically important difference in COPD. Lancet Respir. Med..

