# Supplementary material for: Effect of tiotropium and olodaterol on symptoms and patient-reported outcomes in patients with COPD: results from four randomised, double-blind studies
Source: NPJ Prim Care Respir Med. 2017 Feb 2;27:7. doi: 10.1038/s41533-016-0002-x (PMC5434778; doi:10.1038/s41533-016-0002-x)
Supplement: Supplementary file 1 — Supplementary Material [file 41533_2016_2_MOESM1_ESM.docx]

**Supplementary materials**

**Table S1.** Demographics and baseline characteristics (treated population).

|  | **OTEMTO**^®^ **(*n*=1,621)** | **TONADO**^®^ **(*n*=5,162)** |
| --- | --- | --- |
| Male, *n* (%) | 987 (60.9) | 3,762 (72.9) |
| Mean ± SD age, years | 64.7 ± 8.4 | 64.0 ± 8.3 |
| Mean ± SD duration of COPD, years | 7.8 ± 5.9 | 6.5 ± 5.9 |
| Smoking status, *n* (%)  Ex-smoker  Current smoker | 858 (52.9) 763 (47.1) | 3,254 (63.0) 1,908 (37.0) |
| Smoking history ± SD, pack-years | 49.4 ± 25.4 | 46.2 ± 25.5 |
| Mean (SD) pre-bronchodilator screening  FEV_1_, L | 1.35 (0.49) | 1.20 (0.49) |
| Mean ± SD baseline post-bronchodilator lung function  FEV_1_, L  % of predicted FEV_1_ | 1.54 ± 0.50 55.1 ± 12.8 | 1.37 ± 0.51  50.0 ± 15.3 |
| Mean (SD) change from pre- to post-bronchodilator  FEV_1_, L  FEV_1_/FVC, % | 0.19 (0.15)  50.32 (10.42) | 0.17 (0.15)  44.96 (11.67) |
| Post-bronchodilator % of predicted FEV_1_, *n* (%)^a^  50–<80% (GOLD 2)  30–<50% (GOLD 3)  <30% (GOLD 4) | 1,042 (64.3) 570 (35.2) 8 (0.5) | 2,588 (50.1) 1,989 (38.5) 581 (11.3) |
| Baseline pulmonary medication, *n* (%)  Any  ICS  LAMA  SAMA  LABA  SABA | 1,240 (76.5) 608 (37.5) 560 (34.5) 124 (7.6) 629 (38.8) 818 (50.5) | 4,107 (79.6) 2,446 (47.4) 1,840 (35.6) 665 (12.9) 2,393 (46.4) 2,079 (40.3) |

Abbreviations: COPD, chronic obstructive pulmonary disease; FEV_1_, forced expiratory volume in 1 s; FVC, forced vital capacity; GOLD, Global initiative for chronic Obstructive Lung Disease; ICS, inhaled corticosteroid; LABA, long-acting β_2_-agonist; LAMA, long-acting muscarinic antagonist; SABA, short-acting β-agonist; SAMA, short-acting muscarinic antagonist; SD, standard deviation.
^a^GOLD stage at screening.

**Table S2.** Baseline values for dyspnoea index focal score and SGRQ total score in OTEMTO^®^ and TONADO^®^.

|  | **OTEMTO**^®^ | | **TONADO**^®^ | |
| --- | --- | --- | --- | --- |
|  | **Patients, *n*** | **Mean (SD) BDI** **focal score** | **Patients, *n*** | **Mean (SD) BDI** **focal score** |
| Tiotropium + olodaterol 5/5 µg Tiotropium + olodaterol 2.5/5 µg Tiotropium 2.5 µg Tiotropium 5 µg Placebo Olodaterol 5 µg | 402 402 –– 403 402 –– | 6.6 (2.1) 6.5 (2.0) –– 6.4 (2.0) 6.5 (2.1)  –– | 1,015 1,010 1,015 1,005 –– 1,016 | 6.5 (2.2) 6.5 (2.2) 6.5 (2.2) 6.5 (2.2) –– 6.6 (2.1) |
|  | **Patients, *n*** | **Mean (SE)  SGRQ total score** | **Patients, *n*** | **Mean (SE)  SGRQ total score** |
| Tiotropium + olodaterol 5/5 µg Tiotropium + olodaterol 2.5/5 µg Tiotropium 2.5 µg Tiotropium 5 µg Placebo Olodaterol 5 µg | 393 394 –– 384 370 –– | 42.24 (0.87) 42.60 (0.85) –– 42.81 (0.87) 42.62 (0.92) –– | 979 990 960 955 –– 954 | 44.23 (0.57) 43.84 (0.57) 43.30 (0.57) 43.30 (0.59) –– 42.85 (0.59) |

Abbreviations: BDI, Baseline Dyspnoea Index; SD, standard deviation; SE, standard error; SGRQ, St George’s Respiratory Questionnaire.

**Table S3.** Responder analysis of TDI and SGRQ total score over 12 (OTEMTO^®^) and 24 weeks (TONADO^®^) of treatment.

|  | **OTEMTO**^®^ **(*n*=1,542)^a^** | | | **TONADO**^®^ **(*n*=4,928)^b^** | | |
| --- | --- | --- | --- | --- | --- | --- |
|  | **Odds ratio (SE)** | **95% CI** | ***P*-value** | **Odds ratio (SE)** | **95% CI** | ***P*-value** |
| **TDI responders, treatment differences** | |  |  |  |  |  |
| Tiotropium + olodaterol 5/5 µg  *vs.* olodaterol 5 µg  *vs.* tiotropium 5 µg  *vs.* placebo | –– 1.683 (0.244) 3.297 (0.513) | –– 1.267–2.235 2.430–4.472 | –– 0.0003 <0.0001 | 1.312 (0.118) 1.190 (0.108)  –– | 1.099–1.566 0.997–1.420 –– | 0.0026 0.0546 –– |
| Tiotropium + olodaterol 2.5/5 µg  *vs.* olodaterol 5 µg  *vs.* tiotropium 2.5 µg  *vs.* placebo | –– –– 2.786 (0.433) | –– –– 2.055–3.777 | –– –– <0.0001 | 1.333 (0.120) 1.200 (0.108)  –– | 1.117–1.592 1.005–1.432 –– | 0.0014 0.0439 –– |
| **SGRQ responders, treatment differences** | | |  |  |  |  |
| Tiotropium + olodaterol 5/5 µg  *vs.* olodaterol 5 µg  *vs.* tiotropium 5 µg  *vs.* placebo Tiotropium + olodaterol 2.5/5 µg  *vs.* olodaterol 5 µg  *vs.* tiotropium 2.5 µg  *vs.* placebo | –– 1.559 (0.226) 2.353 (0.354)  –– –– 1.871 (0.282) | –– 1.174–2.070 1.752–3.160  –– –– 1.393–2.513 | –– 0.0022 <0.0001  –– –– <0.0001 | 1.670 (0.153) 1.426 (0.131) ––  1.405 (0.128) 1.157 (0.105) –– | 1.395–1.999 1.192–1.706 ––  1.175–1.679 0.969–1.383 –– | <0.0001 0.0001 ––  0.0002 0.107 –– |

Abbreviations: CI, confidence interval; SE, standard error; SGRQ, St George’s Respiratory Questionnaire; TDI, Transition Dyspnoea Index.
^a^Number of patients contributing to the SGRQ responder analyses in OTEMTO^®^ *n*=1,541.
^b^Number of patients contributing to the SGRQ responder analyses in TONADO^®^ *n*=4,838.

**Table S4.** Deteriorator analysis of TDI and SGRQ total score over 12 (OTEMTO^®^) and 24 weeks (TONADO^®^) of treatment.

|  | **OTEMTO**^®^ **(*n*=1,542)^a^** | | | **TONADO**^®^ **(*n*=4,928)^b^** | | |
| --- | --- | --- | --- | --- | --- | --- |
|  | **Odds ratio (SE)** | **95% CI** | ***P*-value** | **Odds ratio (SE)** | **95% CI** | ***P*-value** |
| **TDI deteriorators, treatment differences** | |  |  |  |  |  |
| Tiotropium + olodaterol 5/5 µg  *vs.* olodaterol 5 µg  *vs.* tiotropium 5 µg  *vs.* placebo | –– 0.827 (0.196) 0.354 (0.075) | –– 0.520–1.315 0.233–0.537 | –– 0.4211 <0.0001 | 0.892 (0.129) 0.775 (0.110)  –– | 0.672–1.185 0.587–1.023 –– | 0.4317 0.0717 –– |
| Tiotropium + olodaterol 2.5/5 µg  *vs.* olodaterol 5 µg  *vs.* tiotropium 2.5 µg  *vs.* placebo | –– –– 0.301 (0.067) | –– –– 0.195–0.465 | –– –– <0.0001 | 0.863 (0.126) 0.804 (0.116)  –– | 0.648–1.149 0.606–1.067 –– | 0.3132 0.1302 –– |
| **SGRQ deteriorators, treatment differences** | | |  |  |  |  |
| Tiotropium + olodaterol 5/5 µg  *vs.* olodaterol 5 µg  *vs.* tiotropium 5 µg  *vs.* placebo Tiotropium + olodaterol 2.5/5 µg  *vs.* olodaterol 5 µg  *vs.* tiotropium 2.5 µg  *vs.* placebo | –– 0.696 (0.125) 0.503 (0.088)  –– –– 0.621 (0.105) | –– 0.490–0.988 0.357–0.709  –– –– 0.447–0.864 | –– 0.0428 <0.0001  –– –– 0.0047 | 0.704 (0.086) 0.715 (0.088) ––  0.930 (0.108) 0.877 (0.101) –– | 0.554–0.895 0.562–0.909 ––  0.740–1.168 0.700–1.096 –– | 0.0042 0.0062 ––  0.5323 0.2560 –– |

Abbreviations: CI, confidence interval; SE, standard error; SGRQ, St George’s Respiratory Questionnaire.
^a^Number of patients contributing to the SGRQ deteriorator analyses in OTEMTO^®^ *n*=1,541.
^b^Number of patients contributing to the SGRQ deteriorator analyses in TONADO^®^ *n*=4,838.

**Table S5.** TDI focal score and SGRQ total score over 12 (OTEMTO^®^) and 24 weeks (TONADO^®^) of treatment.

|  | **OTEMTO**^®^ **(*n*=1,542)^a^** | | | **TONADO**^®^ **(*n*=4,928)^b^** | | |
| --- | --- | --- | --- | --- | --- | --- |
|  | **Adjusted mean (SE)** | **95% CI** | ***P*-value** | **Adjusted mean (SE)** | **95% CI** | ***P*-value** |
| **TDI focal score, treatment comparison** | |  |  |  |  |  |
| Tiotropium + olodaterol 5/5 µg  *vs.* olodaterol 5 µg  *vs.* tiotropium 5 µg  *vs.* placebo | –– 0.594 (0.190) 1.623 (0.193) | –– 0.220–0.967 1.245–2.000 | –– 0.0019 <0.0001 | 0.420 (0.135) 0.356 (0.135) –– | 0.155–0.684 0.092–0.619 –– | 0.0019 0.0082 –– |
| Tiotropium + olodaterol 2.5/5 µg  *vs.* olodaterol 5 µg  *vs.* tiotropium 2.5 µg  *vs.* placebo | –––– –––– 1.611 (0.193) | –––– –––– 1.232–1.989 | –––– –––– <0.0001 | 0.416 (0.135) 0.290 (0.134) –––– | 0.152–0.681 0.027–0.554 –––– | 0.0020 0.0307 –––– |
| **SGRQ total score, treatment comparisons** | |  |  |  |  |  |
| Tiotropium + olodaterol 5/5 µg  *vs.* olodaterol 5 µg  *vs.* tiotropium 5 µg  *vs.* placebo Tiotropium + olodaterol 2.5/5 µg  *vs.* olodaterol 5 µg  *vs.* tiotropium 2.5 µg  *vs.* placebo | –––– -2.097 (0.701) -4.668 (0.710)  –––– –––– -3.846 (0.711) | –––– -3.471, -0.723 -6.060, -3.276  –––– –––– -5.240, -2.451 | –––– 0.0028 <0.0001  –––– –––– <0.0001 | -1.693 (0.553) -1.233 (0.551) ––––  -1.031 (0.552) -0.456 (0.548) –––– | -2.778, -0.608 -2.313, -0.153 ––––  -2.113–0.052 -1.531–0.618 –––– | 0.0022 0.0252 ––––  0.0620 0.4051 –––– |

Abbreviations: CI, confidence interval; SE, standard error; SGRQ, St George’s Respiratory Questionnaire; TDI, Transition Dyspnoea Index.
^a^Number of patients contributing to the SGRQ analyses in OTEMTO^®^ *n*=1,541.
^b^Number of patients contributing to the SGRQ analyses in TONADO^®^ *n*=4,837.
